# Mpox outbreak in the Netherlands, 2022: public health response, characteristics of the first 1,000 cases and protection of the first-generation smallpox vaccine

**DOI:** 10.2807/1560-7917.ES.2023.28.12.2200772

**Published:** 2023-03-23

**Authors:** Catharina E van Ewijk, Fuminari Miura, Gini van Rijckevorsel, Henry JC de Vries, Matthijs RA Welkers, Oda E van den Berg, Ingrid HM Friesema, Patrick R van den Berg, Thomas Dalhuisen, Jacco Wallinga, Diederik Brandwagt, Brigitte AGL van Cleef, Harry Vennema, Bettie Voordouw, Marion Koopmans, Annemiek A van der Eijk, Corien M Swaan, Margreet JM te Wierik, Tjalling Leenstra, Eline Op de Coul, Eelco Franz, the Dutch Mpox Response Team, Members of the Dutch Mpox Response Team, Birgit van Benthem, Diederik Brandwagt, Annemiek A van der Eijk, Hanna Bos, Colette van Bokhoven-Rombouts, Lian Bovée, Chantal P Rovers, Brigitte van Cleef, Alje P van Dam, Rik van Dael, Jaap van Dissel, Pauline Ellerbroek, Catharina van Ewijk, Eelco Franz, Corine GeurtsvanKessel, Joke van der Giessen, Hannelore Götz, Josette Häger, Susan van den Hof, Elske Hoornenborg, Putri Hintaran, Jorgen de Jonge, Rosa Joosten, Marion Koopmans, Kevin Kosterman, Jente Lange, Tjalling Leenstra, André J Meeske, Liesbeth Mollema, Eline Op de Coul, Demi Reurings, Gini van Rijckevorsel, Helma Ruijs, Corien M Swaan, Linda Smid, Gregorius J Sips, Sacha F de Stoppelaar, Albert Vollaard, Bettie Voordouw, Harry Vennema, Henry JC de Vries, Karin Ellen Veldkamp, Klaartje Weijdema, Geert Westerhuis, Margreet JM te Wierik, Matthijs Welkers, Toos Waegemaekers, Jacco Wallinga, Paul Zantkuijl

**Affiliations:** 1 Centre for Infectious Disease Control, National Institute for Public Health and the Environment (RIVM), Bilthoven, the Netherlands; 2 European Programme for Intervention Epidemiology Training (EPIET), European Centre for Disease Prevention and Control (ECDC), Solna, Sweden; 3 Centre for Marine Environmental Studies, Ehime University, Matsuyama, Japan; 4 Department of Infectious Diseases, Public Health Service of Amsterdam, Amsterdam, the Netherlands; 5 Amsterdam UMC location University of Amsterdam, Department of Dermatology, Amsterdam, the Netherlands; 6 Amsterdam Institute for Infection and Immunology, Infectious Diseases, Amsterdam, the Netherlands; 7 Centre for Sexual Health, Department of Infectious Diseases, Public Health Service of Amsterdam, Amsterdam, the Netherlands; 8 Amsterdam Institute for Global Health and Development, Amsterdam, the Netherlands; 9 Amsterdam UMC location AMC, University of Amsterdam, Department of Medical Microbiology and Infection Prevention, Amsterdam, the Netherlands; 10 Department of Biomedical Data Sciences, Leiden University Medical Centre, Leiden, the Netherlands; 11 Department of Viroscience, Erasmus Medical Centre, Rotterdam, the Netherlands

**Keywords:** Mpox, outbreak, the Netherlands, zoonoses, MSM

## Abstract

In early May 2022, a global outbreak of mpox started among persons without travel history to regions known to be enzootic for monkeypox virus (MPXV). On 8 August 2022, the Netherlands reported its 1,000th mpox case, representing a cumulative incidence of 55 per million population, one of the highest cumulative incidences worldwide. We describe characteristics of the first 1,000 mpox cases in the Netherlands, reported between 20 May and 8 August 2022, within the context of the public health response. These cases were predominantly men who have sex with men aged 31–45 years. The vast majority of infections were acquired through sexual contact with casual partners in private or recreational settings including LGBTQIA+ venues in the Netherlands. This indicates that, although some larger upsurges occurred from point-source and/or travel-related events, the outbreak was mainly characterised by sustained transmission within the Netherlands. In addition, we estimated the protective effect of first-generation smallpox vaccine against moderate/severe mpox and found a vaccine effectiveness of 58% (95% CI: 17–78%), suggesting moderate protection against moderate/severe mpox symptoms on top of any possible protection by this vaccine against MPXV infection and disease. Communication with and supporting the at-risk population in following mitigation measures remains essential.

**Key public health message**

**What did you want to address in this study?**

In May 2022, a global outbreak of mpox started (unlike previous outbreaks) among people who had not travelled to regions where monkeypox virus is known to circulate. We wanted to describe the public health response in the Netherlands and the characteristics of the cases to explore which population is at highest risk. We also explored whether the smallpox vaccine available globally before 1978 protects against moderate/severe mpox symptoms.

**What have we learnt from this study?**

International alerts and communication to clinicians led to rapid detection of the first mpox case in the Netherlands. The vast majority of infections occurred in men who (also) have sex with men, aged 31–45 years, and were acquired through sexual contact with casual partners in private or recreational settings including LGBTQIA+ venues in the Netherlands. People vaccinated against smallpox in the 1970ies or before are likely to be protected against moderate/severe mpox symptoms.

**What are the implications of your findings for public health?**

There is no or little indication of transmission through other than direct (sexual) contact. International alerts are important for rapid diagnosis and response. Public health control measures and communication should be aimed at the population at highest risk, who should be included in the design of such outreach programmes. The effect of currently used smallpox vaccines, different from the old vaccine, needs to be researched.

## Background

In early May 2022, the United Kingdom reported cases of monkeypox virus (MPXV) infection among people without travel history to regions known to be enzootic for MPXV such as West and Central Africa [1]. Within weeks, multiple cases were reported in Europe, North-America and Australia [2-4]. Unlike previous reported outbreaks of mpox in the African region and the United States, almost all cases were men who (also) have sex with men (MSM), particularly those with multiple sexual partners.

## Outbreak detection

The first mpox case in the Netherlands was identified at a sexual health clinic (SHC) in Amsterdam after international alerts (EpiPulse, Early Warning and Response System) and confirmed on 20 May 2022. The Netherlands reported the 1,000th mpox case on 8 August 2022, representing a cumulative incidence rate of 55 per million population, one of the highest rates worldwide after Spain (104/million) and Portugal (69/million) at that time, and with 31,112 confirmed mpox cases reported worldwide [5].

More than half (57%) of the Dutch population have never been exposed to orthopoxviruses and can be considered immunologically naïve, as the Netherlands stopped the first-generation smallpox vaccination campaign in 1974 and infections with orthopoxviruses are rare [6,7]. Worldwide, the World Health Organization stopped the smallpox vaccination campaign in 1977. In 1980, smallpox was declared eradicated [8]. Individuals who have been vaccinated with the first-generation smallpox vaccine (which required one dose for complete vaccination) might, however, still benefit from cross-protection against (severe) mpox through MPXV-neutralising antibodies [9,10].

Here we describe the characteristics of the first 1,000 mpox cases in the Netherlands and the public health response, and we provide estimates of protection against moderate/severe mpox symptoms offered by the first-generation smallpox vaccine.

## Methods

### Case detection

Individuals who presented with symptoms suggestive of mpox according to the possible/probable case definition (Box 1) to an SHC or general practitioner (GP) or otherwise were referred and notified to the regional public health service (PHS) for (additional) mpox diagnostics and public health measures (see: Data Collection; Public Health Response).

Box 1 **- Clinical and epidemiological criteria for classification of mpox cases, the Netherlands, as at 8 August 2022**

**Confirmed case**

A person with a laboratory-confirmed MPXV infection (PCR-positive for orthopoxvirus with or without additional MPXV confirmation by sequencing or MPXV-specific PCR).

**Probable case**

A person with skin lesions consistent with mpox on (a part of) the body with symptom onset after 1 March 2022, and/or with complaints consistent with proctitis (including anal pain) that occurred after 1 March 2022, and optionally one or more systemic symptoms^a^ consistent with mpox,

AND one or more of the following criteria:

- contact with a confirmed or probable case of mpox 21 days before symptom onset,
- a man who (also) has sex with men,
- a female partner of a man who (also) has sex with men,
- a person (regardless of sexual orientation) who indicates having had multiple sexual contacts, anonymous or not, or paid for (e.g. at sex parties) 21 days before symptom onset.

**Possible case**

A person with skin lesions consistent with mpox on (a part of) the body with symptom onset after 1 March 2022, and/or with complaints consistent with proctitis (including anal pain) that occurred after 1 March 2022, and optionally one or more systemic symptoms^a^ consistent with mpox,

AND without an epidemiological link with a person who has clinically suspected or confirmed varicella, and where an infection with another known causative agent of similar skin appearance, such as herpes zoster, (primary) herpes simplex, primary or secondary syphilis, is considered unlikely.

MPXV: monkeypox virus.
^a^ Fever (> 38.5 °C), headache, myalgia, backpain, malaise, (usually painful) lymphadenopathy (localised or generalised).

### Laboratory methods

The MPXV laboratory confirmation was performed on pharyngeal and/or skin lesion(s) and/or anal swabs collected either into virus transport medium or on dry or e-swabs. Samples were tested by real-time PCR. Diagnostic protocols (12 diagnostic laboratories) were validated and based either on pan-orthopox real-time PCR with subsequent MPXV detection through sequence analysis or an MPXV-specific PCR [11-13].

### Definitions

Cases were reported as possible, probable and confirmed cases (Box 1). Their contacts were categorised as high-, medium- and low-risk exposure (Box 2). Confirmed mpox cases were categorised as mild or moderate/severe mpox to allow calculation of the vaccine effectiveness (VE) of the first-generation smallpox vaccine against moderate/severe disease. Cases with mild mpox were defined as those experiencing up to two systemic symptoms (e.g. lymphadenopathy, headache, excluding fever) and with skin lesions on up to one body location (head, limbs, oral, trunk or peri-anal/genital). Cases with moderate/severe mpox were defined as those experiencing three or more systemic symptoms (including potential fever) and/or with skin lesions on at least two body locations and/or hospitalisation for mpox. Country of origin was categorised according to the definition from Statistics the Netherlands: the Netherlands, Türkiye, Morocco, the Netherlands Antilles, Surinam and Aruba, ‘other Western’ country which refers to any country on the European continent (excluding Türkiye), North-America, Oceania or Indonesia as well as Japan, and ‘other non-Western’ countries refers to all other countries [14]. People at high risk of MPXV exposure were defined as individuals who engaged in group sex, sex on premises (lesbian gay bisexual transgender queer intersex and asexual and other (LGBTQIA+) venues and saunas) in the Netherlands or abroad, or who were a contact of a confirmed mpox case 21 days before symptom onset.

Box 2 **- Categorisation of high-, medium- and low-risk contacts of mpox cases during the outbreak in the Netherlands, as at 8 August 2022**

**High-risk contact:** a person with one or more of the following types of contact with an mpox case during their infectious period:

- any type of sexual contact,
- intensive skin–skin contact (such as hugging, kissing),
- household contact, excluding intensive skin–skin contact and sexual contact,
- unprotected direct contact with an mpox patient and/or contaminated patient material,
- laboratory employees with unprotected exposure accident involving contaminated material.

**Medium-risk contact:** a person with one or more of the following types of contact with an mpox case during their infectious period

- unprotected prolonged (cumulative more than 2 h) face-to-face contact within 1.5 m distance (such as caregivers without a mouth and nose mask, in social situations, including public transport).

**Low-risk contact:** a person with one or more of the following types of contact with an mpox case during their infectious period

- unprotected short (cumulative less than 2 h) face-to-face contact within 1.5 m distance (such as caregivers without PPE),
- fellow airline travellers with a journey time (more than 8 h) within 1.5 m distance (1–2 seats around the index),
- social contact short (cumulative less than 2 h) face-to-face contact within 1.5 m distance.

**No risk:** a person with one or more of the following types of contact with an mpox case during their infectious period

- caregivers (including laboratory staff) with full PPE: direct contact with an mpox patient and/or contaminated patient material,
- caregivers and social contacts with unprotected exposure at more than 1.5 m distance (regardless of duration).

PPE: personal protective equipment.

### Data collection

Data were collected as part of epidemiological routine surveillance based on obligatory notification of suspected and confirmed cases. After notification to the PHS, a public health team member contacted the case to collect information on demographics including age, sex, medical history, sexual orientation, symptoms, date of symptom onset, hospitalisation and details on source (e.g. risk behaviour and potential source(s) of infection in the 21 days before symptom onset) and contact tracing (numbers of high-, medium- and low-risk contacts). To ensure notification of mpox cases from the PHS to the National Institute for Public Health and the Environment (RIVM), a questionnaire was included in the national surveillance system for notifiable diseases (OSIRIS) for collection of the gathered demographical, clinical and epidemiological information on cases. We extracted for analysis data on mpox cases reported to the RIVM from 20 May until 8 August 2022 from the OSIRIS database.

### Statistical analysis

We described the demographical and epidemiological characteristics of mpox cases. The reporting delay (days) between symptom onset and notification as a confirmed mpox case was calculated. Based on the calculated reporting delay, we estimated the daily reported confirmed cases by date of symptom onset by correcting for under-reporting (i.e. nowcasting) [15].

The VE of the first-generation smallpox vaccine against moderate/severe mpox was calculated among individuals born before 1978 by comparing the odds of first-generation smallpox vaccination between persons with moderate/severe mpox and those with mild mpox. We calculated the VE as 1 minus the odds ratio (OR). The crude OR and 95% confidence intervals (CI) were calculated using logistic regression analyses. Individuals who had received Imvanex (Bacarian Nordif A/S, Kvistgård, Denmark), the third-generation smallpox vaccine approved in the European Union, as post-exposure prophylaxis (PEP) (n = 40) were excluded from VE analyses due to its likely influence on symptom development. In addition, VE was estimated adjusting for age (44–50, 51–55 and ≥ 56 years). Statistical analyses were conducted using R version 4.0.2.

## Results

From confirmation of the first mpox patient on 20 May until 8 August 2022, 1,928 individuals were tested for MPXV: 1,086 (56%) probable and 842 (44%) possible cases. On 8 August, results were pending for 122 persons, 46 probable and 76 possible cases, who were excluded from analyses. Overall, 1,000 of 1,806 (55%) individuals tested positive and 806 (45%) negative for MPXV. Test-positivity was 87% (902/1,040) among probable and 13% (98/766) among possible cases.

The 1,000 confirmed mpox cases had symptom onset dates between 27 April and 2 August 2022 (Figure). Cases were predominately detected via SHC (460/986; 47%), GP (355/986; 36%) and through contact tracing (60/986; 6%). For 14 cases the route of healthcare access was unknown. Most cases (536/1,000; 54%) were reported by the PHS of Amsterdam region, followed by The Hague (85/1,000; 8%) and Rotterdam (60/1,000; 6%) region. Within 1 week after detection of the first case in Amsterdam, six of 25 PHS regions in the Netherlands had reported one or more mpox cases, 20 of 25 within 1 month, and all 25 PHS regions within 2 months.

**Figure:**
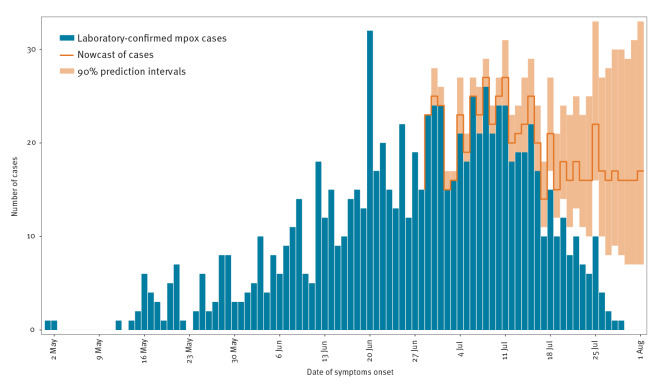
Laboratory-confirmed mpox cases by date of symptom onset, the Netherlands, 20 May–1 August 2022 (n = 894), and nowcast of confirmed cases by date of onset until 1 August

The reporting delay, measured from time of symptom onset to reporting as a confirmed mpox case in OSIRIS, was on average 12 days (median: 10 days) days, with an average delay measured from symptom onset to testing and notification as a suspected case of 6 days (median: 5 days) The number of reported cases by date of symptom onset reached a peak in the first half of July 2022.

### Demographic characteristics

Of all 997 cases, 10 were women and 987 (99%) were men of whom 935 (95%) identified as MSM (Table 1; denominators deviate due to missing data). Median age was 37 years (interquartile range (IQR): 31–45, range: 9–77). Fifty-seven per cent (511/893) were born in the Netherlands, followed by 19% (173/893) in non-Western, 18% (159/893) in other Western countries and 6% (50/893) in Morocco, Türkiye or the Netherlands Antilles, Aruba and Surinam. In total, 187 of 882 (21%) were living with HIV infection, 56 of 882 (6%) reported a concomitant sexually transmitted infection (STI), and 265 of 811 (33%) were on HIV pre-exposure prophylaxis (HIV PrEP) medication. Thirteen per cent (126/948) had received the first-generation smallpox vaccine before 1978. Overall, 227 of 678 (33%) reported known contact (predominantly high-risk) with an mpox case ≤ 21 days before symptom onset, and 18% of them (40/227) had received Imvanex PEP. Cases reported a mean of two high-risk and one medium-risk contacts ranging from 0 to 100 and 0 to 99 contacts, respectively. 

**Table 1:** Demographic characteristics of laboratory confirmed mpox cases in the Netherlands, 20 May–8 August 2022 (n = 1,000)

Categories	Mpox cases	
	n	%
Total	1,000	100
Age group (in years)		
0–17	1	0.1
18–30	241	24
31–40	387	39
41–50	214	21
> 50	156	16
Unknown	1	NA
Sex at birth		
Female	10	1
Male	987	99
Unknown	3	NA
Gender		
Female	7	1
Male	830	98
Transgender man	1	0.1
Transgender woman	1	0.1
Other	4	0.5
Unknown	157	NA
Sexual orientation		
Sex with men	897	94
Sex with men and women	38	4
Sex with women	19	2
Other	2	0.2
Not applicable	2	0.2
Unknown	42	NA
Country of origin		
The Netherlands	511	58
The Netherlands Antilles, Aruba and Surinam	44	5
Morocco	4	0.4
Türkiye	2	0.2
Other Western countries^a^	159	18
Non-Western countries^a^	173	19
Unknown	107	NA
Place of residence		
Amsterdam	536	54
Rest of the Netherlands	464	46
First-generation smallpox vaccination		
No	822	87
Yes	126	13
Unknown	52	NA
Comorbidity		
No	539	61
Yes	343	39
Unknown	118	NA
Type of comorbidity		
HIV infection	187	21
Immunodeficiency other than HIV	13	2
Other STI	56	6
Liver disease	4	0.5
Diabetes mellitus	5	0.6
Cardiovascular disease	16	2
Chronic lung disease	12	1
Kidney disease	4	0.5
Malignancy	1	0.1
Other	62	7
Medication use^b^		
No	336	41
Yes	476	59
Unknown	188	NA
Type of medication		
HIV medication	168	21
HIV PrEP	265	33
Infection (-related) medication^c^	102	13
Contact with a mpox case^d^		
No	452	67
Yes	227	33
Unknown	321	NA
Received Imvanex PEP		
No	869	96
Yes	40	4
Unknown	91	NA
Healthcare worker		
No	863	94
Yes	55	6
Unknown	82	NA
High-risk contacts		
Mean number reported (min–max)	2 (0–100)	
Unknown	122 (NA)	
Medium-risk contacts		
Mean number reported (min–max)	1 (0–99)	
Unknown	216 (NA)	

HIV: human immunodeficiency virus; NA: not applicable; PEP: post-exposure prophylaxis; PrEP: pre-exposure prophylaxis; STI: sexually transmitted infection.

^a^ As defined in [14].

^b^ Including medication against infections such as antibiotics or anti-HIV mediation, immunosuppressants, antiacids, anti-cholesterol drugs, chemotherapy, and HIV PrEP.

^c^ Other than HIV medication, such as antibiotics.

^d^ High- and/or medium-risk contact(s) within 21 days before symptom onset.

Epidemiological data on women and heterosexual men were often lacking (because of unclear exposure or unwillingness to disclose details). Of 10 women (including one transgender person), mostly detected via GP, five reported contact with a case ≤ 21 days before symptom onset. Of 19 male heterosexuals, mostly detected through SHC or GP, two reported contact with a case ≤ 21 days before symptom onset.

### Clinical characteristics

In total 850 of 991 (86%) mpox cases reported systemic symptoms, 914 of 991 (92%) skin lesions (Table 2; denominators deviate due to missing data). Of the 968 cases, 796 (82%) reported both systemic symptoms and skin lesions, 54 (6%) only systemic symptoms and 118 (12%) only skin lesions. Of cases experiencing both skin lesions and systemic symptoms with dates of onset reported (632/796), 50% (316/632) reported that skin lesions had developed up to 9 days (median: 2 days) after the first systemic symptom(s), 24% (154/632) reported that skin lesion(s) and systemic symptom(s) had developed on the same day, and 26% (162/632) reported that the first skin lesion(s) had developed up to 13 days (median: 3 days) before systemic symptoms.

**Table 2:** Clinical characteristics of mpox cases, the Netherlands, 20 May–8 August 2022 (n = 1,000)

Categories	Mpox cases	
	n	%
Total	1,000	100
Symptoms		
Only skin lesion(s)	118	12
Only systemic symptom(s)	54	6
Both lesions and systemic symptoms	796	82
Unknown	32	NA
Presence of systemic symptoms		
No	141	14
Yes	850	86
Unknown	9	NA
Type of systemic symptoms		
Fever	521	53
Headache	322	32
Myalgia	257	26
Malaise	268	27
Lymphadenopathy	371	37
Itching	134	14
Proctitis	179	18
Diarrhoea and/or vomiting	45	5
Coughing	43	4
Respiratory symptoms, other	81	8
Backpain	70	7
Other	33	3
Number of systemic symptoms reported		
1–2	405	49
≥ 3	425	51
Unknown	170	NA
Onset of systemic symptoms and skin lesions, if both reported		
Systemic symptoms before lesions	316	50
Systemic symptoms and lesions same day	154	24
Systemic symptoms after lesions	162	26
Unknown	368	NA
Number of days skin lesions started after onset of systemic symptoms^a^		
Median; IQR (95^th^ percentile)	2 days; 2 days (9 days)	
Number of days systemic symptoms started after onset skin lesions^b^		
Median; IQR (95^th^ percentile)	3 days; 2 days (13 days)	
Presence of skin lesions		
No	77	8
Yes	914	92
Unknown	9	NA
Number of body locations with skin lesions		
1	341	37
≥ 2	573	63
Body location with skin lesions		
Head	312	34
Oral	105	11
Trunk	350	38
Limbs	469	51
Genital and/or perianal	684	75
Genital	469	51
Perianal	305	33
Number of types of lesions present		
1	536	59
≥ 2	376	41
Type of lesions		
Maculopapular	286	31
Vesicular	536	59
Pustular	420	46
Crusts	107	12
Other	45	5
Hospitalisation		
No	942	99
Yes	11	1
Unknown	47	NA
Severity of mpox^c^		
Mild mpox	215	26
Moderate/severe mpox	618	74
Unknown	167	NA

IQR: interquartile range; NA: not applicable.

^a^ Based on 316 cases with skin lesion onset after systemic symptoms.

^b^ Based on 162 cases with systemic symptom onset after the onset of skin lesions.

^c^ Mild mpox: cases experiencing no more than two systemic symptoms (e.g. lymphadenopathy, headache, excluding fever) and skin lesions on no more than one body location (e.g. head, limps, trunk, peri-anal/genital). Moderate/severe mpox: cases experiencing three or more systemic symptoms and/or skin lesions on two or more body locations and/or hospitalisation for mpox.

Fifty-one per cent (425/830) reported three or more systemic symptoms, and 63% (573/912) lesions on at least two different body locations. Only 43 of 991 (4%) reported coughing and 81 of 991 (8%) other respiratory symptoms. Reporting any respiratory symptoms did not correlate with the presence of oral lesions: of cases reporting respiratory symptoms, 14% reported oral lesions compared with 11% of cases not reporting respiratory symptoms (p = 0.6). Twenty-six percent (215/833) of cases had mild mpox and 74% (711/991) moderate/severe mpox symptoms. One infection was detected in a child and details have been published [16].

### Transmission routes and exposure

Sexual contact was the dominant reported route of transmission (822/865; 95%) (Table 3: denominators deviate due to missing data). Transmission most probably occurred in a family/home setting (417/977; 43%), other recreational settings such as house parties (217/977; 22%) or LGBTQIA+ nightlife in the Netherlands (201/977, 21%). Most cases (577/920; 63%) had not travelled abroad ≤ 21 days before symptom onset, contrary to the beginning of the outbreak when 26 of 39 cases reported travel in May compared with 192 of 526 (37%) in July and 15 of 53 in August. Those who travelled visited mostly European countries (316/343; 92%), predominantly Germany (n = 99), Spain (n = 94) and Belgium (n = 44). Twenty-three per cent (194/861) reported participation in sex-on-premises and 44% (315/708) in group sex 21 days before symptom onset. Sexual partners were mostly casual (708/771; 92%).

**Table 3:** Reported transmission routes of infection and behavioural characteristics of mpox cases, the Netherlands, 20 May–8 August 2022 (n = 1,000)

Categories	Mpox cases	
	n	%
Total	1,000	100
Most likely transmission route		
Sexual contact^a^	822	95
Direct unprotected contact	15	2
Household	5	0.6
Prolonged face-to-face contact	20	2
Other	3	0.4
Unknown	135	NA
Most likely setting(s) for transmission^b^		
LGBTQIA+ venues/events NL	201	21
LGBTQIA+ venues/events abroad	134	14
General nightlife/events NL	42	4
General nightlife/events abroad	35	4
Other recreational settings	217	22
Family/home setting	417	43
Healthcare	1	0.1
Work	10	1
School	1	0.1
Travel	6	0.6
Unknown	23	NA
Number of times travelled abroad		
Did not travel	577	63
1 time	287	31
≥ 2 times	56	6
Unknown	80	NA
Cases who reported travel among all monthly cases		
May	26/39	67
June	110/302	36
July	192/526	37
August	15/53	28
Countries that cases visited^b^		
European	316	34
Non-European	36	84
Visited sex on premises venue(s) abroad		
No	695	76
Yes, only visited	75	8
Yes, participated in sexual activities	150	16
Unknown	80	NA
Visited venues in the Netherlands		
No	539	63
1 time	258	30
≥ 2 times	64	7
Unknown	139	NA
Type of entertainment venues NL^b^		
LGBTQIA+ sex venues/places	144	17
LGBTQIA+ bars	64	7
Private settings	27	3
Other nightlife	117	14
Sex on premises venue(s) in the NL		
No	539	63
Only visited	128	15
Participated in sexual activities	194	23
Number of sexual activities reported for cases with sexual contact as most likely route of transmission		
1–2	286	46
≥ 3	335	54
Unknown	336	NA
NA	43	NA
Type of sexual activity reported^b^		
Anal	530	85
Oral	432	70
Kissing	317	51
Intimate skin-skin contact	306	49
Oral-anal	95	15
Vaginal	9	1
Sharing sex toys	14	2
Masturbating	50	8
Unknown/NA	378	NA
Participated in group sex		
No	393	56
Yes	315	44
Unknown/NA	292	NA
Type of sexual partner		
Casual partner	708	92
Steady partner	63	8
Unknown/NA	229	NA

LGBTQIA+: lesbian gay bisexual transgender queer intersex and asexual and other; NL: the Netherlands; NA: not applicable.

^a^ Including mucosal and intensive skin-skin contact.

^b^ More than one answer was possible.

### Smallpox vaccine effectiveness against moderate/severe mpox

The VE of the first-generation smallpox vaccine against moderate/severe mpox was estimated at 59% (95% CI: 24–78%). After adjustment for age, the VE remained similar (58%; 95% CI: 15–80%) (Table 4). A sensitivity analysis with a more stringent classification of mild and moderate/severe mpox showed a VE estimate of 50% (95% CI: −10 to 78%) but with wide confidence interval due to a small sample size.

**Table 4:** Vaccine effectiveness of the first-generation smallpox vaccine against moderate/severe mpox, the Netherlands, 20 May–8 August 2022 (n = 177)

Vaccination status	Number of mild mpox cases	Number of severe mpox cases	Total cases	Crude vaccine effectiveness	95% CI	Adjusted vaccine effectiveness^a^	95% CI
Unvaccinated	23	51	74	Reference		Reference	
Vaccinated	54	49	103	59%	24–78	58%	12–80
Sensitivity analysis							
Unvaccinated	23	18	41	Reference		Reference	
Vaccinated	54	21	75	50%	−10 to 78	47%	−31 to 79

CI: confidence interval.

^a^ Adjusted for age in 44–50, 51–55 and ≥ 56 years.

Mild mpox: cases experiencing no more than two systemic symptoms (e.g. lymphadenopathy, headache, excluding fever) and skin lesions on no more than one body location (e.g. head, limps, trunk, peri-anal/genital) only. Moderate/severe mpox: cases experiencing three or more systemic symptoms (including fever) AND/OR skin lesions on at least two body locations and/or hospitalisation for mpox. Classifications used in sensitivity analysis: Mild mpox: cases experiencing no more than two systemic symptoms (e.g. lymphadenopathy, headache, excluding fever) and skin lesions on no more than one body location (e.g. head, limps, trunk, peri-anal/genital) only. Moderate/severe mpox: cases experiencing three or more systemic symptoms (including fever) AND skin lesions on at least two body locations and/or hospitalisation for mpox.

### Public health response

In view of the increasing number of mpox cases in neighbouring European countries early May 2022, an mpox response team (RT) was convened on 18 May at the Centre for Infectious Disease Control (CIb) of the RIVM. The RT consisted of experts from the CIb, representatives of regional PHS, the external reference laboratory (Viroscience Erasmus Medical Centre), the medical expert working group on sexual health and STI (WASS), Soa Aids Nederland – a Dutch STI policy and prevention foundation for professionals and the public – and infectious disease specialists. The RT provided scientific advice (on request of the Ministry of Health, (MoH)) on risk assessment and classification, notifiable disease status, diagnostics, infection prevention and control measures regarding cases and contacts including risk communication and pre- and post-exposure vaccination. After the notification of the first mpox case on 20 May and on advice of the RT, the MoH declared mpox a notifiable disease in group A on 20 May. This group A status entails centralised coordination of the response and swift public health actions, including mandatory notification within 24 h by treating physicians and laboratories to the PHS of both suspected (possible/probable) as well as confirmed cases, case isolation, source and contact tracing and the ability to quarantine contacts [17]. After healthcare professionals notified a suspected case, a public health team member contacted the case to arrange diagnostic testing and advise self-isolation and hygiene measures. If mpox diagnosis was confirmed, the PHS informed and monitored high- and medium-risk contacts by telephone for development of symptoms up to 21 days after their last exposure. In addition, high-risk contacts were offered the Imvanex vaccine as PEP – preferably administered within 4 and up to 14 days after first exposure.

Initially, contacts who had sexual, intimate skin-to-skin or household contact (high-risk contact; Box 2) with mpox cases were asked to self-quarantine for 21 days. However, on 24 June, a national council of experts (Deskundigenberaad) advised to replace this quarantine measure with the advice to refrain from intimate (and sexual) contact during the 21-day monitoring period. Reasons for this policy change were the increasing evidence that direct skin-to-skin or sexual contact were the dominant transmission routes and challenges during contact tracing, such as hesitancy to reveal contact information of (sexual) contacts because of the potential consequences of prolonged quarantine. A second policy change was made on 7 July, when 2-dose Imvanex vaccination schemes were offered as pre-exposure vaccination (mpox PrEP) to individuals at high risk of mpox to curb transmission. Transgender persons and MSM were eligible if they (i) received HIV PrEP (or were on a HIV PrEP waiting list) via SHC or GP, (ii) were living with HIV and screened for hepatitis C as a proxy for increased risk of STI or (iii) were known at the SHC or GP to be at increased risk of STI, including MSM sex workers [18]. Individuals who had received a first-generation smallpox vaccination in the past required only one dose of Imvanex vaccine for completion of mpox PrEP. The vaccination campaign started on 25 July in two public health regions with the highest mpox incidence (Amsterdam and The Hague), later followed by all regions in the Netherlands. Individuals who were eligible received an invitation by post and/or email, and/or telephone.

### Risk communication

The RT also coordinated risk communication towards the at-risk population, professionals and the general public. The RIVM provided information on mpox to the general public (e.g. on disease characteristics, symptoms and prevention) through the RIVM website and (social) media. Professionals (e.g. medical microbiologists, infectious disease physicians, SHC and PHS professionals) were informed through the mpox guideline, and were regularly updated on clinical characteristics, (new) diagnostics, control measures and the epidemiological situation through direct messaging services [19-21].

Soa Aids Nederland coordinated the mpox communication campaign targeting the MSM community. Communication materials including posters, flyers and digital content were developed to improve knowledge on and raise awareness for mpox (e.g. symptoms, partner notification, self-isolation, vaccination and other risk-reducing measures). These materials were distributed to all PHS, HIV treatment centres and LGBTQIA+ venues across the country, including gay clubs, saunas and other (sex) venues, and social events such as Gay Prides. LTBTQIA+ club owners and event organisers were informed about mpox, transmission risks and prevention measures (such as hygiene guidelines) for sex-on-premises. A manual was developed for PHS to guide outreach control measures and to start a dialogue about mpox with the LGBTQIA+ community [22]. Online campaigns included information on mpox (prevention) on websites, Facebook and Instagram accounts from organisations targeting LGBTQIA+ communities (such as Man-tot-Man from the PHS of Amsterdam, PrEP-NU, several regional PHS website and COC the Netherlands – an organisation advocating the LGBTQIA+ rights, as well as gay-dating apps such as Grindr and Recon). Information and real-life stories were shared through podcasts. In addition, Soa Aids Nederland facilitated and provided financial support to the LGBTQIA + community to organise their own mpox webinar (‘Het Grote Monkeypox Informatie Webinar’ on 3 August 2022).

## Discussion

We describe the mpox outbreak in the Netherlands, which was part of an international outbreak, and the Dutch public health response [2,4]. The first 1,000 mpox cases, reported between 20 May and 8 August 2022, were predominantly MSM aged 31–45 years, similar to outbreaks in other countries [1,23-25]. The vast majority of infections were acquired through sexual contact with casual partners at family/home or recreational settings including LGBTQIA+ venues in the Netherlands. This indicates that, although some larger upsurges were related to point-source and/or travel-related events, the outbreak was mainly characterised by sustained transmission within the country.

International alerts and communication to relevant clinicians led to rapid detection of the first mpox case in the Netherlands. A retrospective study showed no evidence of transmission before May 2022 [26]. Within 1 day of the first confirmed case, the Dutch MoH declared mpox a notifiable disease in group A. This allowed for a nationally coordinated response, notification of both suspected and confirmed cases and the ability to quarantine contacts, essential measures when aiming for disease elimination with little prior information on disease characteristics such as dominant transmission route and severity. As the outbreak continued, there was little evidence of indirect or respiratory transmission and, combined with signals from the LGBTQIA+ community on low intention to isolate/quarantine, it was important to scale down initial stringent measures as soon as evidence allowed, to prevent potential counter-productive effects of the measures.

Although 33% of the cases reported contact with another case ≤ 21 days before symptom onset, only 6% were detected though contact tracing, and information on risk behaviour and sexual (anonymous) contacts was often lacking. Communicating with and supporting the at-risk population in following mitigation measures will therefore remain essential. Besides rapid communication efforts towards the LGBTQIA+ community, there was active involvement of e.g. gay clubs and sauna owners in developing educational materials and outreach programmes.

Previous international reports show that mpox is more severe in elderly people, pregnant women, children and immunocompromised people, with mortality rates between 1% and 10% [27,28]. In the current outbreak, with ongoing sexual person-to-person transmission, few hospitalisations and deaths have been reported internationally, and it seems that fewer than one in 1,000 cases die from mpox-related complications [27-29]. This difference in morbidity and mortality could be explained by bias in the populations involved, as the current circulation is not seen in children, pregnant women and rarely in immunocompromised people, or by selection bias related to health system constraints in Africa with additionally possible overreporting of severe cases. Another reason could be the specific strain involved or differences in the mode of transmission as few cases reported respiratory symptoms (12%) and there was no or little indication of indirect transmission.

Most mpox cases reported both systemic symptoms and skin lesions and for about half of these cases, skin lesions developed up to 9 days (median: 2 days) after systemic symptoms onset. In addition, 6% of cases reported only systemic symptoms. This highlights the importance of screening for MPXV in persons at high-risk of mpox, even in the absence of skin lesions, as well as clinical follow-up after a positive test. Delay in testing or ending self-isolation too soon increases the risk of further transmission. 

Few studies have looked at VE of the first-generation smallpox vaccine against (moderate/severe) mpox, and there is limited evidence on its immunogenicity against mpox. An observational study from 1988 showed an estimated VE of 85% against mpox disease among household contacts, and a recent study showed the presence of MPXV-neutralising antibodies in those with historic smallpox vaccination [10,30,31]. We did not have adequate data to allow estimation of VE of first-generation smallpox vaccines against mpox infection. However, we found among those born before 1978 a VE of 58% (95% CI: 12–80%) against moderate/severe mpox. This suggests moderate protection against mpox symptoms on top of any possible protection by the first-generation vaccine against MPXV infection or disease.

Although only 13% of all cases had received a first-generation smallpox vaccine and thus no protection is expected for the majority of cases, this result can support policymakers in allocating scarce vaccines. In the Netherlands Imvanex is available as mpox PrEP for populations at high risk to prevent mpox infection and curb transmission. Those who were vaccinated with the first-generation vaccine, received only one dose of Imvanex vaccine for complete mpox PrEP rather than two. It is uncertain if our VE estimate of the first-generation smallpox vaccine is transferable to the third-generation smallpox vaccine (Imvanex) presently in use. Whereas the first-generation vaccine contained infectious vaccinia virus, the third-generation vaccine contains a further attenuated, non-replicating vaccinia virus and is therefore less immunogenic than the first-generation vaccine [32]. A recent study suggests the third-generation vaccine induces fewer antibodies with cross-reactivity to mpox than the first-generation vaccine [10]. VE estimates of Imvanex against MPXV infection, mpox symptoms and/or duration of illness could impact public health control measures such as vaccination strategies and isolation.

There are several limitations to our study. Our data were cross-sectional and data on disease progression were not available. Also, differences in the time point of first contact with the PHS between mild and moderate/severe cases can bias VE estimates, e.g. mild cases were contacted earlier by the PHS than moderate/severe cases, and symptoms developing after that contact might not be registered. However, within our population for VE analysis, there was no significant difference in time between symptom onset and the first PHS contact between mild and moderate/severe mpox cases: mild cases were contacted on (median) Day 6 (IQR: Day 5) and moderate/severe cases on (median) Day 5 (IQR: Day 5) after symptom onset (p = 0.28). We used self-reported vaccination status to calculate VE, potentially leading to misclassification. In addition, the classification of mild and moderate/severe disease is somewhat arbitrary and based on combined number(s) and body locations of symptoms. However, disease severity is rather subjective, and people may experience mpox disease to different extent.

Although the outbreak was still ongoing at a low level in September 2022, the number of reported cases by date of symptom onset reached a peak in the first half of July 2022. This could in part be a result of the public health control measures and of acquired immunity from natural infection or vaccination in persons with high risk of MPXV exposure. The full extent of the outbreak, including potential asymptomatic MPXV infections, may have been higher [33]. Serological population studies and case–control studies may provide additional information on the wider extent of exposure and infection.

## Conclusions

The 2022 outbreak of mpox among MSM was mainly characterised by sustained transmission through direct (sexual) contact. International alerts and communication to relevant clinicians led to rapid detection of the first mpox case in the Netherlands. Individuals who received a first-generation smallpox vaccine had a lower probability of developing moderate/severe mpox. Communication with and support of the at-risk population in following mitigation measures will remain essential.

## Note

CvE is a fellow of the ECDC Fellowship Programme, supported financially by the European Centre for Disease Prevention and Control The views and opinions expressed herein do not state or reflect those of ECDC. ECDC is not responsible for the data and information collation and analysis and cannot be held liable for conclusions or opinions drawn.
